# Effect of Broad-Spectrum Hemp Extract on Neurobehavioral Activity on the Immobilization Stress-Induced Model in Sprague Dawley Rats

**DOI:** 10.1155/2023/3425576

**Published:** 2023-06-10

**Authors:** Devanand Shanmugasundaram, James Martin Roza

**Affiliations:** ^1^Compliance and Operations (Toxicology), Vedic Lifesciences Pvt. Ltd., Mumbai, India; ^2^HempRise, 285 Paul Garrett Avenue, Jeffersonville, IN 47130, USA

## Abstract

**Background:**

Broad-spectrum hemp extract is expected to be a promising new intervention for managing stress and anxiety. Research has shown that the cannabinoids found in *Cannabis sativa*, such as cannabidiol (CBD), tetrahydrocannabinol (THC), and cannabigerol (CBG), possess anxiolytic properties that can positively impact mood and stress.

**Methods:**

In the current study, a broad-spectrum, nondetectable THC hemp extract with other minor cannabinoids (broad-spectrum hemp extract) was administered at 28 mg/kg·bw to evaluate its anxiolytic properties. This was performed using various behavioural models and biomarkers for oxidative stress. In addition, a 300 mg/kg·bw of Ashwagandha root extract was also incorporated to compare its effects on relieving stress and anxiety.

**Results:**

The decreased levels of lipid peroxidation were measured in broad-spectrum hemp extract (36 nmol/ml), Ashwagandha (37 nmol/ml), and induction control (49 nmol/ml) treated groups of animals. The levels of 2-AG decreased in the broad-spectrum hemp extract (1.5 ng/ml), Ashwagandha (1.2 ng/ml), and induction control (2.3 ng/ml) treated groups of animals. The levels of FAAH decreased in broad-spectrum hemp extract (1.6 ng/ml), Ashwagandha (1.7 ng/ml), and induction control (1.9 ng/ml) treated groups of animals. The levels of catalase increased in broad-spectrum hemp extract (35 ng/ml), Ashwagandha (37 ng/ml), and induction control (17 ng/ml) treated groups of animals. Similarly, increased levels of glutathione were found in broad-spectrum hemp extract (30 ng/ml), Ashwagandha (27 ng/ml), and induction control (16 ng/ml) treated groups of animals.

**Conclusion:**

Based on the results of this study, it can be concluded that broad-spectrum hemp extract inhibited the biomarkers for oxidative stress. Also, certain behavioural parameters showed improvements with respect to both the ingredient administered groups.

## 1. Introduction

Stress and anxiety have long been associated with various health issues, including hypertension, obesity, cardiovascular diseases, and mental health [1]. Elevated stress levels can trigger higher levels of cortisol which, over an extended period, can cause the narrowing of the arteries, leading to high blood pressure. This eventually may result in vessel damage and plaque build-up. It can also lead to a myriad of other health issues, ranging from immune suppression, gastrointestinal problems, sleeplessness, and weight gain. Therefore, it is important to find ways to manage stress through diet, exercise, lifestyle changes, meditation, or supplementation before a drug intervention, if possible.

The passage of the Farm Bill in 2018 (Agriculture Improvement Act) [2] decriminalized the cultivation of hemp and allowed broader use of its derivatives. The flower of the *Cannabis* plant, primarily known as a source of the psychoactive compound delta-9-tetrahydrocannabinol, has interested researchers. This is due to the substantial number of other compounds, including over one hundred cannabinoids, four hundred terpenes, and numerous other phenolic compounds. Before the passage of the Farm Bill, a great deal of the research, dated back to 1940, was limited to only those who were either licensed to study a controlled substance or done outside USA. Beginning with the discovery of cannabinol (CBN) in 1940 and the subsequent discovery of cannabidiol (CBD) a couple of years later, interest in cannabinoids has been steadfast and has led to many discoveries. One of the most significant was the revelation that all mammals possess an endocannabinoid system and that cannabinoids can profoundly affect the human body's immune and nervous systems [3]. CBD has been shown to interact with cell receptors such as serotonin 5-HT, vanilloid, CB2, and G-coupled protein [4–6] that regulate fear and anxiety-related behaviours. This research has led to new and novel ways to attenuate stress using CBD instead of benzodiazepines or other drugs. In numerous preclinical studies, CBD has been proven to have antiepileptic, antioxidant, anti-inflammatory, antipsychotic, anxiolytic, and antidepressant properties. In clinical trials, CBD was proven safe, well-tolerated, and effective in several types of seizure disorders and childhood epilepsy. In addition, subjects with Parkinson's disease (PD) have reported improved sleep and quality of life with CBD [7]. Literature evidence suggests CBD has shown some initial ability to improve cancer tumours and pain and acts as an anti-inflammatory, analgesic, antiarthritic, anti-Alzheimer, antidepressant, and antidiabetic [8–10].

Therefore, the key objective of this trial was to assess the impact a broad-spectrum CBD extract would have on rats, as exemplified by their behaviour and oxidative stress parameters compared to one of the most commonly used dietary supplements for stress, Ashwagandha [11].

## 2. Methods and Materials

### 2.1. Chemicals, Reagents, and Kits

Kits for Rat Lipid Peroxide ELISA (Lot No. 202202001), Rat Catalase ELISA (Lot No. 202202001), Rat Glutathione peroxidase ELISA (Lot No. 202202001), Rat 2-Arachidonoylglycerol ELISA (Lot No. 202202001), and Rat fatty acid amide hydrolase ELISA (Lot No. 202202001) were purchased from Bioassay Technology Laboratory, United Kingdom. All the chemicals and reagents used for the trial were of analytical grade, procured from India.

### 2.2. Preparation

HempBroad™ (Batch No. HBCB200826), a light yellow to amber-coloured oil with a manufacturing date 26^th^ August 2020 and expiry date 26^th^ August 2025, was manufactured using an ethanol/heptane solvent extraction process and standardized to 80% cannabidiol and 1% minor cannabinoids with a THC level of less than 0.3% and stored in a tight glass/aluminium/plastic/stainless sell container in a dark, cool, refrigerated area away from direct heat. Ashwagandha (Batch No. 21/S018), a light, yellowish-brown powder with a manufacturing date January 2021 and expiry date December 2023, was stored in a store in original, sealed containers at room temperature. The broad-spectrum hemp extract and Ashwagandha dosages were prepared before each dosing based on the individual body weights of the animals. The daily oral administration of the broad-spectrum hemp extract was 28 mg·kg^−1^·bw and of Ashwagandha was 300 mg·kg^−1^·bw, per the study design.

### 2.3. Experimental Animals and Welfare

The study was conducted in India by following the guidelines specified by the Committee for the Purpose of Control and Supervision of Experiments on Animals (CPCSEA), the Institutional Animal Ethics Committee (IAEC), and as per the Standard Operating Procedures (Approval No. LBPL/IAEC/073/10/2021). Adult male Sprague Dawley rats (∼6-7 weeks old, mean body weight of ∼160–180 g) were imbibed into the study. Animals were procured from a CPCSEA-approved vendor in compliance with ethical practices. These are in accordance with the guidelines for animal care and are accredited by the American Association for Accreditation of Laboratory Animal Care (AAALAC), USA. The animals were subjected to veterinary examination and allowed to acclimatize to the laboratory environment for five days. Animals were provided access to feed and water *ad libitum* as per experimental conditions (temperature 23 ± 2°C; relative humidity 50–69%, and 12 h alternate light/dark cycle with 12–15 cycles/hour of air change). Reverse osmosis water and commercial pellet feed were provided *ad libitum* for 4 weeks.

### 2.4. Experimental Design

The study was conducted using 24 adult male SD rats that were randomly divided into four groups (*n* = 6): Group 1 (G1): induction control and no treatment were given; Group 2 (G2): placebo medium chain triglycerides (MCT oil); Group 3 (G3): broad-spectrum hemp extract (28 mg·kg^−1^·bw) for 4 weeks; and Group 4 (G4): Ashwagandha (300 mg·kg^−1^·bw) for 4 weeks. Dose formulations were administered to the specific treatment groups for 28 consecutive days. On the 28^th^ day, all the animals were immobilized for 6 h by taping all four limbs on a board using zinc oxide tape. At the end of the immobilization, the skin regions were moistened with acetone to minimize pain or discomfort. The rats were kept in an animal cage with soft bedding in the experimental room. After immobilization, behavioural and biochemical parameters were determined.

### 2.5. Oral Administration

The required test and reference material quantity was weighed and mixed with MCT oil. The prepared dose formulation was administered to respective animal groups by oral gavage using a 3 mL disposable syringe attached to a 16/18 oral gavage. Test item administration was performed on continuous stirring using a magnetic stirrer to maintain homogeneity. The dose volume was 10 mL/kg body weight for all animals. The dose volume administered to each animal was calculated based on the most recent body.

### 2.6. In-Life Observations

The animals were monitored weekly for body weight gain and feeding patterns. Additionally, any clinical signs were recorded daily during the treatment period. This also included recording any changes in gait, posture, and response to handling and the presence of clonic or tonic movements, stereotypes (repetitive circling), or bizarre behaviour. Blood samples were collected with the capillary tube by retro-orbital plexus puncture under isoflurane anaesthesia. All the animals were euthanized on terminal sacrifice using the carbon dioxide (CO_2_) asphyxiation method.

### 2.7. Biomarkers (Oxidative Parameters)

All the blood samples were allowed to stand for complete clotting and then centrifuged at 2500 rpm for 15 minutes at 4°C. The collected serum was separated for oxidative stress markers, *viz*., lipid peroxidation (LPO), catalase, glutathione peroxidation (GPX), 2-AG (2-arachidonoylglycerol). Fatty acid amide hydrolase (FAAH) was determined by the enzyme-linked immunosorbent assay (ELISA) method as per the Bioassay Technology Laboratory protocol.

### 2.8. Lipid Peroxidation (LPO)

Before the procedure, all reagents (standard solutions and sample) were brought down to room temperature. Strips were inserted in the frames for use. The unused strips were stored at 2–8°C. Next, 50 *µ*l of the standard solution, 40 *µ*l of the sample, and 10 *µ*l of anti-LPO antibody were added. Furthermore, 50 *µ*l of streptavidin-HRP was added to the sample and standard wells and mixed well. The plate was covered with a sealer and incubated at 37°C for 60 minutes. Postincubation, the plates were washed with wash buffer 5 times. Wells were soaked with 300 *µ*l of wash buffer for 1 minute for each wash. Plates were blotted onto the absorbent material. 50 *µ*l of substrate solution A was added to each well and followed by substrate solution B. Plates were incubated with a fresh sealer at 37°C for 10 minutes in the dark. Finally, 50 *µ*l of stop solution was added to each well and observed for a change of blue to yellow colour. Optical density (OD value) was immediately measured using a microplate reader at 450 nm.

### 2.9. Catalase

Before the procedure, all reagents (standard solutions and sample) were brought down to room temperature. Strips were inserted in the frames for use. The unused strips were stored at 2–8°C. Next, 50 *µ*l of the standard solution, 40 *µ*l of the sample, and 10 *µ*l of anti-CAT antibody were added. Furthermore, 50 *µ*l of streptavidin-HRP was added to the sample and standard wells and mixed well. The plate was covered with a sealer and incubated at 37°C for 60 minutes. Postincubation, the plates were washed with wash buffer 5 times. Wells were soaked with 300 *µ*l of wash buffer for 1 minute for each wash. Plates were blotted onto the absorbent material. 50 *µ*l of substrate solution A was added to each well and followed by substrate solution B. Plates were incubated at 37°C for 10 minutes in the dark. Finally, 50 *µ*l of stop solution was added to each well and observed for a change of blue to yellow colour. Optical density (OD value) was immediately measured using a microplate reader at 450 nm.

### 2.10. Glutathione Peroxidation (GPx)

Before the procedure, all reagents (standard solutions and sample) were brought down to room temperature. Strips were inserted in the frames for use. The unused strips were stored at 2–8°C. Next, 50 *µ*l of the standard solution, 40 *µ*l of the sample, and 10 *µ*l anti-GPX antibody were added. Furthermore, 50 *µ*l of streptavidin-HRP was added to the sample and standard wells and mixed well. The plate was covered with a sealer and incubated at 37°C for 60 minutes. Postincubation, the plates were washed with wash buffer 5 times. Wells were soaked with 300 *µ*l of wash buffer for 1 minute for each wash. Plates were blotted onto the absorbent material. 50 *µ*l of substrate solution A was added to each well and followed by substrate solution B. Plates were incubated at 37°C for 10 minutes in the dark. Finally, 50 *µ*l of stop solution was added to each well and observed for a change of blue to yellow colour. Optical density (OD value) was immediately measured using a microplate reader at 450 nm.

### 2.11. 2-AG (2-Arachidonoylglycerol)

Before the procedure, all reagents (standard solutions and sample) were brought down to room temperature. Strips were inserted in the frames for use. The unused strips were stored at 2–8°C. Next, 50 *µ*l of the standard solution, 40 *µ*l of the sample, and 10 *µ*l anti-2-AG antibody were added. Furthermore, 50 *µ*l of streptavidin-HRP was added to the sample and standard wells and mixed well. The plate was covered with a sealer and incubated at 37°C for 60 minutes. Postincubation, the plates were washed with wash buffer 5 times. Wells were soaked with 300 *µ*l of wash buffer for 1 minute for each wash. Plates were blotted onto the absorbent material. 50 *µ*l of substrate solution A was added to each well and followed by substrate solution B. Plates were incubated at 37°C for 10 minutes in the dark. Finally, 50 *µ*l of stop solution was added to each well and observed for a change of blue to yellow colour. Optical density (OD value) was immediately measured using a microplate reader at 450 nm.

### 2.12. FAAH (Fatty Acid Amide Hydrolase)

Before the procedure, all reagents (standard solutions and sample) were brought down to room temperature. Strips were inserted in the frames for use. The unused strips were stored at 2–8°C. Next, 50 *µ*l of the standard solution, 40 *µ*l of the sample, and 10 *µ*l anti-FAAH antibody were added. Furthermore, 50 *µ*l of streptavidin-HRP was added to the sample and standard wells and mixed well. The plate was covered with a sealer and incubated at 37°C for 60 minutes. Postincubation, the plates were washed with wash buffer 5 times. Wells were soaked with 300 *µ*l of wash buffer for 1 minute for each wash. Plates were blotted onto the absorbent material. 50 *µ*l of substrate solution A was added to each well and followed by substrate solution B. Plates were incubated at 37°C for 10 minutes in the dark. Finally, 50 *µ*l of stop solution was added to each well and observed for a change of blue to yellow colour. Optical density (OD value) was immediately measured using a microplate reader at 450 nm.

### 2.13. Statistical Analysis

Data were expressed as mean ± standard deviation (SD). Statistical analysis was performed using One-way analysis of variance (ANOVA) and GraphPad Prism software (Version 7.04, San Diego, CA, USA), followed by Dunnett's test. This was done to compare the treatment group with the respective groups. The value for *P* < 0.05 was considered statistically significant.

## 3. Results and Discussion

### 3.1. Neurobehavioral Observations

No clinical signs, mortality, or morbidity were observed in any treatment groups during the study; even the body weights and feed consumption were unaltered during the experimental period.

The elevated-plus maze model is one of the behavioural assays used to test a material's antianxiety effect. Open and closed arms are considered to induce the same exploratory drive; therefore, avoiding open arms is considered an outcome of higher levels of fear [12]. To prove this hypothesis, the broad-spectrum hemp extract (G3) and standardized Ashwagandha extract (G4) treated animals exhibited more entries, time spent, number of crosses, and time required in the closed arm as compared to the G1 and G2 groups.

The open-field test is behavioural research's most frequently used method. The method involves analyzing locomotion, anxiety, and stereotypical behaviours in rodents [13]. For example, stressed animals prefer staying close to the walls and moving towards the periphery, exhibiting anxiety-like behaviour. Conversely, animals with lower anxiety levels tend to spend more time in the central area of the box. The trial showed that the broad-spectrum hemp extract (G3) and standardized Ashwagandha extract (G4) treated animals showed less faecal count, increased time spent in the centre, and increased number of crosses. At the same time, G1 and G2 group animals spent more time in the corner with an increased faecal count.

The despair swim test is one of the widely used assays to study rodent depression-like behaviour [14]. When the animal is placed in a container filled with water in a swim test, it will try to escape. Eventually, it will exhibit immobility that may reflect a measure of behavioural despair. There was a significant decrease in the immobility time of the broad-spectrum hemp extract (G3) and standardized Ashwagandha extract (G4) groups compared to the G1 and G2 group animals.

The light/dark test is based on the characteristic behaviour of the rodents; they are opposed to brightly illuminated areas in response to mild stressors (light) [15]. Broad-spectrum hemp extract (G3) and standardized Ashwagandha extract (G4) treated animals spent less time and fewer entries in the light compartment than the G1 and G2 group animals. Similarly, broad-spectrum hemp extract (G3) and standardized Ashwagandha extract (G4) treated animals spent more time and more entries in the dark compartment than the G1 and G2 group animals (Table 1).

### 3.2. Oxidative Parameters

Anxiety, stress, and depression have a strong oxidative stress component because of increased reactive oxygen species (ROS) generation. These result from increased oxidant production during psychological stress and elevated cortisol levels [16]. Biomarkers such as lipid peroxidation, catalase, and glutathione are good indicators of how stress and anxiety can affect redox processes within the body. In this study, these biomarkers were used to assess the effect of the test materials compared to the induction control group. Additionally, this study also measured the impact the test materials had on the endogenously produced endocannabinoid, 2-arachidonoylglycerol (2-AG), and the enzyme fatty acid amide hydrolase (FAAH), both of which are part of pathways that regulate endocannabinoid signalling in the nervous system and are associated with stress reduction.

Lipid peroxidation is the chain of oxidative degradation reaction of lipids, where free radicals “steal” electrons from the lipids. Lipid peroxidation has been implicated in various diseases and pathological conditions. The trial showed lower levels of lipid peroxidation found in broad-spectrum hemp extract (36 nmol/ml) and standardized Ashwagandha extract (37 nmol/ml) treated animals in comparison with an induction control group (49 nmol/ml).

Catalase is a crucial enzyme that catalyzes hydrogen peroxide to water and oxygen. It mainly protects the cells from oxidative damage by reactive oxygen species (ROS). In our study, the levels of catalase increased in broad-spectrum hemp extract (35 ng/ml) and standardized Ashwagandha extract (37 ng/ml) treated animals in comparison with the induction control group (17 ng/ml).

Glutathione (GSH) is involved in detoxifying both xenobiotic and endogenous compounds. It scavenges oxidants such as superoxide anion, hydroxyl radical, nitric oxide, and carbon radicals. GSH depletion has been strongly associated with diseases and loss of function with ageing. In our current investigation, increased levels of glutathione were found in broad-spectrum hemp extract (30 ng/ml) and standardized Ashwagandha extract (27 ng/ml) treated animals in comparison with the induction control group (16 ng/ml).

2-Arachidonoylglycerol plays a vital role in regulating the circulatory system. In addition, accumulating evidence reveals that 2-AG is involved in various shocks and pathogenesis.

Thus, it may be a novel and an attractive therapeutic target. However, the levels of 2-AG decreased in the broad-spectrum hemp extract (1.5 ng/ml) and standardized Ashwagandha extract (1.2 ng/ml) treated animals in comparison with the induction control group (2.3 ng/ml).

Fatty acid amide hydrolase (FAAH) is an integral membrane enzyme that hydrolyzes the endocannabinoid anandamide and related amidated signalling lipids. Pharmacological inactivation of FAAH produces analgesic, anti-inflammatory, anxiolytic, and antidepressant phenotypes, without showing the undesirable side effects of direct cannabinoid receptor agonists. This indicates that FAAH may be a promising therapeutic target. The levels of FAAH decreased in broad-spectrum hemp extract (1.6 ng/ml) and standardized Ashwagandha extract (1.7 ng/ml) treated animals in comparison with the induction control group (1.9 ng/ml) (Table 2).

Ashwagandha (tradename KSM-66) was chosen as the positive control because it is the most popular dietary supplement to manage stress. In addition, it has been the subject of published research showing that it can attenuate stress [17, 18].

The study aimed to show the impact of HempRise broad spectrum CBD on attenuating anxiety compared with Ashwagandha. The neurotransmitters anandamide (N-arachidonoylethanolamine) and 2-AG (arachidonoylglycerol) were the two main neurotransmitters of interest because they are the only two endocannabinoids that have been generally recognized by the scientific community thus far. While they are other neurotransmitters that are shown to affect receptors such as CB1 and CB2 and other G protein-coupled receptors involved in attenuating anxiety, the purpose of the study was to see how the cannabinoids in the HempRise broad-spectrum test material (CBD and other minor cannabinoids) would impact the body in supporting the body's internal endocannabinoid system.

## 4. Conclusion

Research on cannabidiol has shown it to be a promising new agent in attenuating anxiety, stress, and other related disorders [19, 20]. The increased incidences of stress and anxiety among all age groups and its effects on mental health have drawn scrutiny from health administrators worldwide. Over-reliance on benzodiazepines and their side effects has necessitated new and novel approaches to counter these conditions. Cannabidiol is the most abundant cannabinoid in hemp flower, next to delta-9-tetrahydrocannabinol and a hundred other native cannabinoids, and is responsible in part for the positive health benefits seen in medical marijuana. The amounts of CBD, however, can vary depending on the plant's strain and the amount of THC present. Therefore, a concentrated form of CBD, as seen in broad-spectrum hemp extract without the psychoactive properties of THC, is a promising intervention in dealing with anxiety-related disorders. The results of this study demonstrate CBD's effects in managing ROS caused by environmental stressors by reducing lipid peroxidation and increasing the levels of catalase and glutathione. It also further exemplified its impact on the endocannabinoid system by reducing levels of 2-AG and FAAH. While results from the neurobehavioral tests were equivocal, improvements were seen in the open-field and swim despair tests compared with the induction control group. This study also showed that CBD was comparable to Ashwagandha in modifying the behaviour of the rodents as exhibited in the test results from the open-field and swim despair tests and improving the oxidative stress parameter scores.

## Figures and Tables

**Table 1:** Summary of neurobehavioral parameters.

Parameters	Group		G1	G2	G3	G4
	No. of rats		6	6	6	6
Elevated-plus maze	Time required to enter open arms (in sec)	Mean	24	22	19	18
		SEM	1.6	3.1	2.9	1.8
	Time required to enter closed arms (in sec)	Mean	18	15	11	9.5^*∗∗*^
		SD	1.7	1.5	0.92	1.3
	No. of entries in open	Mean	4.3	4.2	4.0	3.5
	Arms	SD	0.61	0.31	0.58	0.43
	No. of entries in closed arms	Mean	3.2	3.7	4.8^*∗*^	5.5^*∗∗*^
		SD	0.31	0.49	0.31	0.62
	Time spent in open arms (in sec)	Mean	61	47	44	45
		SD	8.2	7.2	6.7	5.1
	Time spent in closed arms (in sec)	Mean	197	217	225^*∗*^	228^*∗*^
		SD	6.9	7.5	6.7	4.6

Open-field test	No. of cross	Mean	6.7	7.7	14^*∗*^	15^*∗*^
		SD	1.2	0.99	2.6	1.8
	Time spent in the corner (in sec)	Mean	248	236	221	230
		SD	8.4	10	7.4	12
	Time spent in centre (in sec)	Mean	25	33	34	37
		SD	3.7	5.6	4.6	7.5
	Faecal count (in no.)	Mean	4.2	3.3	2.3^*∗*^	1.5^*∗∗*^
		SD	0.31	0.71	0.42	0.34

Despair swim test	Immobility time (in sec)	Mean	23	19	16^*∗*^	14^*∗∗*^
		SD	2.3	1.9	0.75	0.95

Light/dark model	Time spent in the light compartment (in sec)	Mean	107	87	72^*∗*^	67^*∗*^
		SD	14	6.9	5.7	7.5
	Time spent in the dark compartment (in sec)	Mean	184	198	221^*∗*^	223^*∗*^
		SD	13	8.5	8.2	8.6
	No. of entries in the light compartment	Mean	4.3	3.8	3.0	3.2
		SD	0.61	0.60	0.26	0.40
	No. of entries in dark compartment	Mean	3.2	3.7	4.5	5.2^*∗*^
		SD	0.31	0.49	0.34	0.65

*Note*. The values are expressed in mean ± SEM. *n* = 6. ^*∗*^*P* < 0.01, ^*∗∗*^*P* < 0.001 as compared to the induction control (G1) group.

**Table 2:** Summary of oxidative parameters.

Parameters	Group	G1	G2	G3	G4
	No. of rats	6	6	6	6
Catalase (ng/ml)	Mean	17	25	35	37
	SEM	11	12	12	9.5

Lipid peroxidation LPO (nmol·ml)	Mean	49	38	36	37
	SEM	11	7.8	7.8	3.1

Glutathione (ng/ml)	Mean	16	24	30	27
	SEM	6.0	11	6.1	5.7

22-AG (2-arachidonoylglycerol) (ng/ml)	Mean	2.3	1.7	1.5	1.2
	SEM	0.79	0.25	0.19	0.16

Fatty acid amide hydrolase (FAAH) (ng/L)	Mean	1.9	1.6	1.6	1.7
	SEM	0.30	0.14	0.17	0.17

*Note*. The values are expressed in mean ± SEM.

## Data Availability

No underlying data were collected or produced in this study.

## References

[1] Yaribeygi H., Panahi Y., Sahraei H., Johnston T. P., Sahebkar A. (2017). The impact of stress on body function: a review. *EXCLI J*.

[2] Govinfo (2018). The 2018 Farm Bill (agricultural improvement act) public law 115-334.

[3] Schurman L. D., Lu D., Kendall D. A., Howlett A. C., Lichtman A. H. (2020). Molecular mechanism and cannabinoid pharmacology. *Handbook of Experimental Pharmacology*.

[4] Izzo A. A., Borrelli F., Capasso R., Di Marzo V., Mechoulam R. (2009). Nonpsychotropic plant cannabinoids: new therapeutic opportunities from an ancient herb. *Trends in Pharmacological Sciences*.

[5] Campos A. C., Moreira F. A., Gomes F. V., Del Bel E. A., Guimaraes F. S. (2012). Multiple mechanisms involved in the large-spectrum therapeutic potential of cannabidiol in psychiatric disorders. *Philosophical Transactions of the Royal Society B: Biological Sciences*.

[6] McPartland J. M., Duncan M., Di Marzo V., Pertwee R. G. (2015). Are cannabidiol and Delta (9) tetrahydrocannabivarin negative modulators of the endocannabinoid system? A systematic review. *British Journal of Pharmacology*.

[7] Elsaid S., Kloiber S., Le Foll B. (2019). Effects of cannabidiol (CBD) in neuropsychiatric disorders: a review of pre-clinical and clinical findings. *Prog Mol Biol Transl Sci*.

[8] Turna J., Syan S. K., Frey B. N. (2019). Cannabidiol as a novel candidate alcohol use disorder pharmacotherapy: a systematic review. *Alcoholism: Clinical and Experimental Research*.

[9] Urits I., Borchart M., Hasegawa M., Kochanski J., Orhurhu V., Viswanath O. (2019). An update of current cannabis-based pharmaceuticals in pain medicine. *Pain Ther*.

[10] Noreen N., Muhammad F., Akhtar B., Azam F., Anwar M. I. (2018). Is cannabidiol a promising substance for new drug development? A review of its potential therapeutic applications. *Critical Reviews in Eukaryotic Gene Expression*.

[11] Pratte M. A., Nanavati K. B., Young V., Morley C. P. J. (2014). An alternative treatment for anxiety: a systematic review of human trial results reported for the Ayuverdic herb ashwagandha (*Withania sominfera*). *Journal of Alternative and Complementary Medicine*.

[12] Rodgers R. J., Dalvi A. (1997). Anxiety, defence and the elevated plus-maze. *Neuroscience and Biobehavioral Reviews*.

[13] Kraeuter A. K., Guest P. C., Sarnyai Z. (2019). The open Field test for measuring locomotor activity and anxiety-like behavior. *Methods in Molecular Biology*.

[14] Yankelevitch-Yahav R., Franko M., Huly A., Doron R. (2015). The forced swim test as a model of depressive-like behavior. *Journal of Visualized Experiments: JoVE*.

[15] Bourin M., Hascoët M. (2003). The mouse light/dark box test. *European Journal of Pharmacology*.

[16] Salim S. (2017). Oxidative stress and the central nervous system. *Journal of Pharmacology and Experimental Therapeutics*.

[17] Chandrasekhar K., Kapoor J., Anishetty S. (2012). A prospective, randomized double-blind, placebo-controlled study of safety and efficacy of a high-concentration full-spectrum extract of ashwagandha root in reducing stress and anxiety in adults. *Indian Journal of Psychological Medicine*.

[18] Akhgarjand C., Asoudeh F., Bagheri A. (2022). Does Ashwagandha supplementation have a beneficial effect on the management of anxiety and stress? A systematic review and meta-analysis of randomized controlled trials. *Phytotherapy Research*.

[19] Skelley J. W., Deas C. M., Curren Z., Ennis J. (2003). Use of cannabidiol in anxiety and anxiety-related disorders. *Journal of the American Pharmacists Association*.

[20] Shannon S., Lewis N., Lee H., Hughes S. (2019). Cannabidiol in anxiety and sleep: a large case series. *The Permanente Journal*.

